# How Team-Level and Individual-Level Conflict Influences Team Commitment: A Multilevel Investigation

**DOI:** 10.3389/fpsyg.2017.02365

**Published:** 2018-01-17

**Authors:** Sanghyun Lee, Seungwoo Kwon, Shung J. Shin, MinSoo Kim, In-Jo Park

**Affiliations:** ^1^School of Business, Hanyang University, Seoul, South Korea; ^2^Korea University Business School, Seoul, South Korea; ^3^The School of Business, Portland State University, Portland, OR, United States; ^4^School of Management, Wuhan University of Science and Technology, Wuhan, China

**Keywords:** individual-level conflict, team-level conflict, task conflict, relationship conflict, team commitment, multilevel analysis

## Abstract

We investigate how two different types of conflict (task conflict and relationship conflict) at two different levels (individual-level and team-level) influence individual team commitment. The analysis was conducted using data we collected from 193 employees in 31 branch offices of a Korean commercial bank. The relationships at multiple levels were tested using hierarchical linear modeling (HLM). The results showed that individual-level relationship conflict was negatively related to team commitment while individual-level task conflict was not. In addition, both team-level task and relationship conflict were negatively associated with team commitment. Finally, only team-level relationship conflict significantly moderated the relationship between individual-level relationship conflict and team commitment. We further derive theoretical implications of these findings.

## Introduction

As modern organizations are increasingly adopting team-based work structure, academics and practitioners have paid huge attention to team dynamics including team conflict (Nesterkin and Porterfield, 2016). Because a team cannot perform well without managing team conflict effectively (de Wit et al., 2012; Bradley et al., 2015), it is critical to understand how team conflict influences team dynamics and its success. Although team commitment has been recognized as one of the critical determinants of team success (Kukenberger et al., 2012; Mathieu and Gilson, 2012; Mahembe and Engelbrecht, 2013), there still has been a paucity of studies investigating the relationship between conflict and team commitment. To answer this call, this study examines the relationships between different types and levels of conflict and team commitment.

Team commitment refers to the relative strength of an individual's identification with and involvement in a particular team. It is likely to increase the team members' (1) beliefs in, and acceptances of, the team's goals and values; (2) willingness to exert considerable effort on behalf of the team; and (3) desire to maintain membership in the team (Bishop and Scott, 2000). While team commitment plays critical roles for team success, intragroup conflict, which is a natural phenomenon in any team setting, may have a significant relationship with team commitment. For example, intragroup conflict can incur interpersonal problems within the team, increasing negative emotions such as tension and anxiety and lowering positive emotions such as team satisfaction (Jehn, 1995; De Dreu and Gelfand, 2008; de Wit et al., 2012; DeChurch et al., 2013), which may result in their weak team commitment. However, given the mixed findings about the effects of conflict on team success (e.g., Jehn et al., 1999; Pelled et al., 1999; de Wit et al., 2012) and some suggested boundary conditions for the relationship (e.g., Bradley et al., 2013, 2015), we cannot simply expect a negative relationship between conflict and team commitment. In this study, we investigate the effects of two different types of conflict (i.e., relationship conflict and task conflict) on the two different levels (i.e., individual- and team-level) on team commitment.

Prior studies have found that resolving relationship conflict among team members is critical for team success as relationship conflict often generates negative consequences on teams. However, the findings regarding the effects of task conflict have been mixed; the results have included negative consequences (Jehn et al., 1999; Lovelace et al., 2001; De Dreu and Weingart, 2003; de Wit et al., 2012), positive consequences (Jehn, 1995; Amason, 1996; Matsuo, 2006; Song et al., 2006; Olson et al., 2007), and no significant consequences on teams (Pelled et al., 1999; de Wit et al., 2012). In addition, while the bulk of conflict research (e.g., Baron, 1991; Amason, 1996; Jehn et al., 1997, 1999; Pelled et al., 1999; Bailey, 2000; Simons and Peterson, 2000; Jehn and Mannix, 2001; Olson et al., 2007) has focused on the different conflict types in the last few decades, research on this issue has largely proceeded with a single-level theory, and research has not clearly specified the level of analysis in theory or measurement (Korsgaard et al., 2008).

Given the lack of research on the relationship between conflict and team commitment, and given the fact that conflict research has neglected the multilevel nature of intragroup conflict, multilevel theorization and verification are necessary to enhance our knowledge of how conflict affects team members. To answer these calls, we adopt a multilevel perspective (Klein and Kozlowski, 2000; Ceschi et al., 2014) by investigating the combined effect of individual-level and team-level conflict on individual attitudes toward the team (i.e., team commitment). In the current study, we first distinguish individual-level conflict and team-level conflict. Then, at these two levels, we clarify the effects of task conflict and relationship conflict on team commitment. In addition, based on social information processing theory (Salancik and Pfeffer, 1978), we examine if the team-level conflict moderates the relation between the individual-level conflict and team commitment. Salancik and Pfeffer (1978) emphasized that an individual's attitude is influenced by the social information about what others think, and thus individuals can acquire their own attitude or behavior by learning the information of the social environment. An important source of information is the individual's “immediate social environment” because it provides cues that individuals use to construct and interpret events. We argue that team-level conflict can be an important source of social information. Because individual team members may collect and use social cues from their team in order to interpret the nature of their individual conflict, we propose that team-level conflict influences how perceived individual conflict affects the attitude toward their team such as team commitment.

Our study contributes to conflict literature in several ways. First, we examine the relationship between conflict and team commitment, which has been rarely studied. It would be interesting to examine how within-team conflict affects individual attitudes such as team commitment. Moreover, because team commitment is important for current and future team success (Hackman, 1987), the investigation of the determinants would be helpful to better understand how to maximize team productivity. Secondly, we investigate the relationship at multiple levels analyzing the multilevel model with hierarchical linear modeling (HLM). The attitudes of team members toward their team are influenced by the team-level conflict as well as each team member's personal experience with other members (i.e., individual-level conflict). However, past studies have mostly focused on the effects of individual-level conflict perceptions on individual-level outcomes and very few studies have examined the effects of team-level conflict on individual-level attitudes. We fill this void by investigating how team-level conflict relates to individual team commitment. Furthermore, with the multilevel approach, we may be able to better understand the multilevel phenomenon of how team conflict influences the individual-level attitude of team commitment. Finally, we theorize and test cross-level interactions between individual-level and team-level conflicts on team commitment. To date, the conflict literature says little about the consequences of the interactional effect of individual- and team-level conflict. Because individual- and team-level conflicts may influence each other, studying this interaction could provide a more robust understanding of how conflict affects a team and its members.

## Theoretical frameworks and hypotheses

### The multilevel nature of intragroup conflict

According to Klein and Kozlowski (2000), organizations are multilevel systems, with individuals nested within groups, and groups nested within organizations. As a result, the entities—individuals, dyads, teams, groups, organizations, and so on—are tied with each other (Klein et al., 1994). Accordingly, certain levels inevitably interact with each other: characteristics of one level have effects throughout levels above and below (Rousseau, 1985). Conflict issues are no exception to this multilevel interaction. In particular, conflict may initially exist only among certain members of a team (Jehn, 1995), but such isolated individual-level conflict can quickly escalate into a team-level conflict with a set of attributes specific to that particular team. In turn, this team-level conflict will influence individual-level conflict, and the behaviors and attitudes of the team's members. In the next section, following Rousseau's (1985) suggestion, we begin with the explicit description of the properties that differentiate individual-level conflict from team-level conflict.

While individual-level conflict refers to the individual's recognition of conflict experienced from firsthand interpersonal interactions with a specific team member(s), team-level conflict refers to the team members' recognition of conflict existing in the team as a whole regardless of the focal individual's involvement. Here, team-level conflict is a set of summary perceptions reflecting an interaction between personal and organizational characteristics (James and Jones, 1974). In addition, individual-level and team-level conflict are distinguished by the reference of the conflict. While individual-level conflict is affected by interpersonal characteristics (i.e., team member reference), team-level conflict is influenced by team characteristics (i.e., work team reference).

### The effect of individual-level conflict on team commitment

Individual-level conflict refers to an interpersonal incompatibility or differing perceptions such as a difference of opinions and/or an unmatched relationship in interacting with others. Individual-level conflict can have both negative and positive consequences on a team and its team members. For example, some studies have reported that individual-level conflict is related to negative consequences because the conflict causes tension and hostility and reduces team member satisfaction and team productivity (March and Simon, 1958; Pondy, 1967; Hackman and Morris, 1975; Farh et al., 2010; de Wit et al., 2012). On the other hand, other studies have found positive consequences of individual-level conflict. For example, it can stimulate innovative thinking and the creation of new ideas (Coser, 1956; Walton, 1969; Deutsch, 1973).

As an attempt to reconcile such contrasting findings, conflict researchers suggested distinguishing within-team conflict into task conflict and relationship conflict. Although the definitions vary (Pinkley, 1990; Priem and Price, 1991; Jehn, 1995; Amason, 1996), they consistently emphasize distinguishing relationship/affective/social-emotional conflict from task/substantive/cognitive/goal-oriented conflict because they lead to different consequences. Here, relationship conflict refers to interpersonal incompatibility and includes tension, annoyance and animosity among team members; while task conflict refers to disagreements among team members regarding the content of their decisions and includes differences in viewpoints, ideas, and opinions (Jehn, 1995). In other words, task conflict is rooted in the substance of the task that a team is undertaking whereas relationship conflict derives from the emotional, affective aspects of the team's interpersonal relations (Guetzkow and Gyr, 1954).

Individual-level task conflict is expected to have significant influence on satisfaction or team commitment. Individual-level task conflict can lead to positive consequences for certain types of tasks such as creative performance and decision quality in top management teams (Schweiger et al., 1989; Jehn, 1995; Simons and Peterson, 2000), in particular via a self-regulated process (e.g., Ceschi et al., 2017). However, according to self-verification theory (Swann et al., 2004), individuals become dissatisfied when their perspectives or solutions are challenged by group members, since it can be interpreted as a negative assessment of their own abilities. Therefore, individual-level task conflict is likely to negatively relate to satisfaction (Jehn, 1995; de Wit et al., 2012). Task conflict may also cause tension and resentment among team members leading to dissatisfaction with the interpersonal interaction (Amason and Schweiger, 1994; Gamero et al., 2008) and decreasing the desire of team members to stay on the team (Schweiger et al., 1986).

In interpersonal relationship conflict, team members are likely to experience interpersonal tension and negative emotions such as anxiety and fear, which leads to a less positive attitude toward the team (Walton and Dutton, 1969; Jehn and Bendersky, 2003; Dijkstra et al., 2005; de Wit et al., 2012). Accordingly, relationship conflict, which is related to nervousness, hatred, and negative self-valuation, is likely to reduce team commitment and increase turnover intention (Elron, 1997; Bayazit and Mannix, 2003; Raver and Gelfand, 2005; Rispens et al., 2007; Jehn et al., 2008; Rispens, 2012).

In sum, both task and relationship conflicts at individual level are expected to be negatively related to team commitment. Accordingly, we hypothesize as follows:

*Hypothesis 1a. Individual-level task conflict will be negatively related to team members' commitment*.
*Hypothesis 1b. Individual-level relationship conflict will be negatively related to team members' commitment*.

### The effect of team-level conflict on team commitment

Team-level conflict is a shared perception amongst the team members (Klein and Kozlowski, 2000). Team-level conflict is not objective and is not an observable concept, but it can be measured by the degree of agreeableness among team members (West et al., 2009). Accordingly, team-level conflict is expected to work as a contextual factor at the team level that can predict individual-level attitudes beyond the effects of the individual-level conflict experience. For example, a team member may perceive high level of team-level conflict even when he or she does not have any interpersonal conflict with team members. In this case, he or she would not want to be involved in a conflict that has already occurred among other group members. That is, he or she may want to stay away from the conflict among other group members. Individual team members are likely to adopt avoidance behavior particularly when they perceive all types of conflict that arise from irreconcilable differences among team members (O'Neill et al., 2013). Avoidance behavior will aggravate differences among group members since they cannot understand the reasons for others' positions (Tjosvold, 2008). Therefore, individuals are not likely to identify with the team or to be committed to the team. In this case, the team member's team commitment is likely to be negatively influenced by the team-level conflict. Team-level conflict is a contextual variable that may globally influence an individual's commitment toward the team. Therefore, regardless of how much conflict each team member has individually with other team members, team-level conflict will have a negative influence on team commitment. With a perception of task or relationship conflict among other members, a team member is likely to have a negative attitude toward the team in the form of nervousness and worry, which will then lower his or her team commitment. Thus, we propose that team-level conflict has incremental effects on team commitment over the effects of individual-level conflict.

*Hypothesis 2a. Holding individual-level conflict constant, team-level task conflict is negatively related to team commitment*.
*Hypothesis 2b. Holding individual-level conflict constant, team-level relationship conflict is negatively related to team commitment*.

### A cross-level interaction between the two different levels of conflict

In addition to examining the incremental effect of team-level conflict on team commitment, our study explores the moderating role of team-level conflict on the relationship between individual-level conflict and team commitment. In particular, we propose that team-level conflict may also affect how a team member reflects his/her perception of an individual-level conflict (e.g., arising from his/her disagreement with other team members) on his/her team commitment. In other words, team-level conflict may affect how team members perceive individual-level disagreements and emotional conflicts with other team members, in turn influencing their general attitude toward their team. Social information processing theory (Salancik and Pfeffer, 1978) helps explaining why this would be the case. Social information processing theory (Salancik and Pfeffer, 1978) argues that an individual's attitude is influenced by social information about what people around them think. In the same vein, we argue that team-level conflict can be an important source of social information so that team-level conflict plays a moderating role in the relationship between individual-level conflict and team commitment. When individuals form attitudes toward their team, they use information gathered from other team members. Via social information processing, the shared perception of team-level conflict is likely to influence how individual team members process their perceptions of the conflict experienced at an individual level and in turn, how they form their attitudes toward their team. For example, when team-level conflict is high, the negative relationship between individual team members' individual-level conflicts and their team commitment is likely to be stronger than when team-level conflict is low. Under a high team-level conflict, members tend to respond to each other with more defensiveness and animosity even when the disagreement is constructive. This intensifies the negative effect of individual-level conflict on their attitude toward their team. On the contrary, when team-level conflict is low, the negative relationship between individual-level conflict and team commitment is likely to be attenuated, because members may regard their individual-level conflict as just their own problems and not that of their team's. In addition, they may take the interpersonal conflict less seriously due to their perception of the team climate as positive.

More specifically, we argue that team-level task conflict is likely to aggravate the negative relationship between individual-level task conflict and team commitment, and that team-level relationship conflict is likely to aggravate the negative relationship between individual-level relationship conflict and team commitment. Drawing on social information theory, our premise is that team-level conflict influences how team members interpret and react to their own individual conflict with another team member(s). Therefore, we propose that team-level conflict is an important feature of a work team's social context, and can enhance or weaken the previously hypothesized effects of the individual level conflict on team commitment.

*Hypothesis 3a. The relationship between individual-level task conflict and an individual's team commitment will be moderated by team-level task conflict*.
*Hypothesis 3b. The relationship between individual-level relationship conflict and an individual's team commitment will be moderated by team-level relationship conflict*.

**Figure 1:**
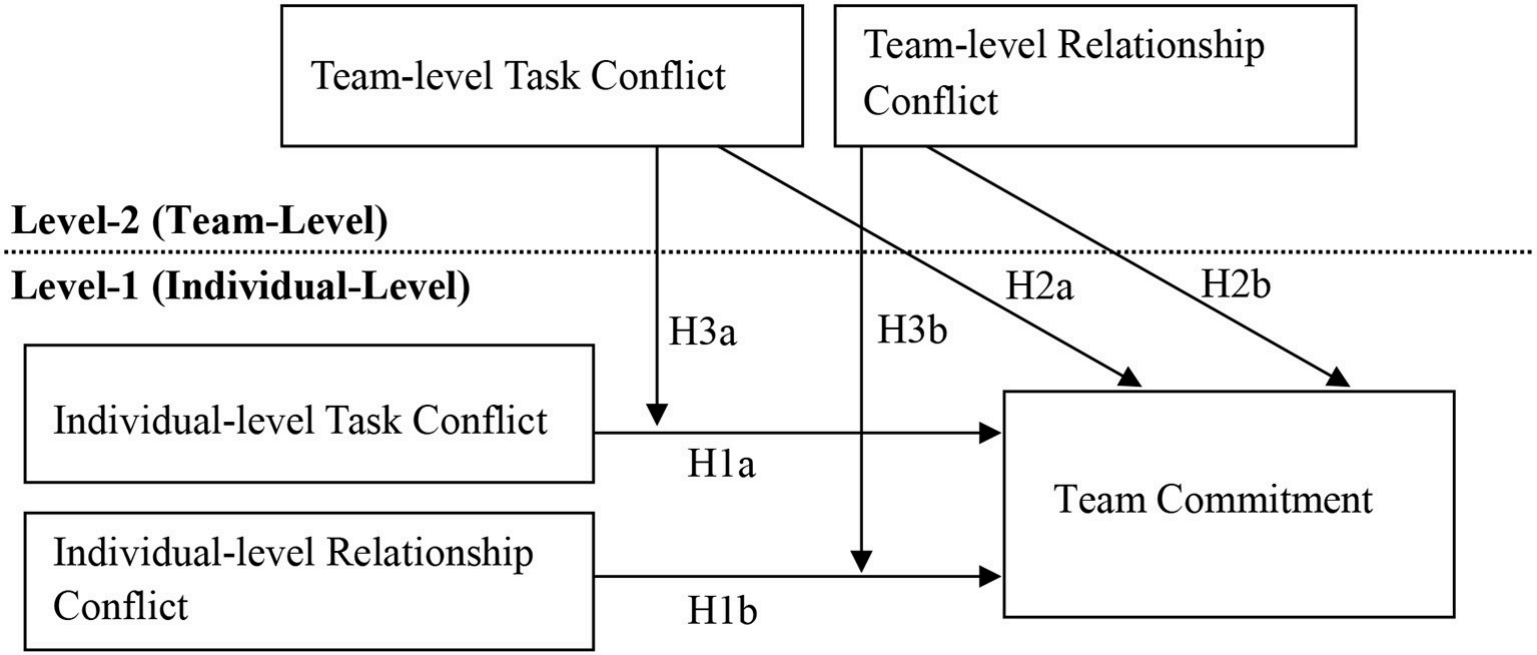
Hypothesized multilevel model of the intragroup conflict.

The model of this study is conceptualized in Figure 1.

## Method

We collected data from 452 employees in 57 branch offices of a private commercial bank in Korea. Each of the branch offices had a registered branch manager who was responsible for business operations. While the size of the branches, in terms of the number of employees and customers, varies a little depending on the location of the branch (e.g., urban or rural), the structures, processes and contents of the work are identical across the branches. In addition, employees interacted with other employees within their own branch on a daily base with task interdependence. Thus, bank branches were conceptualized as defined work teams of interdependent individuals. Typically, each of the bank branches is composed of a branch manager, one or two sub-branch manager (depending on branch size), and bank clerks who perform teller tasks. The members of each branch perform organizationally relevant tasks and share common goals and must coordinate with each other to carry out their tasks. The team-level data were collected from each branch and the individual-level data were collected from individuals within each branch. The response rate of the survey was 52.7 percent. We collected data from branch offices with 3 or more employees (team size in branches ranged from 6 to 22 individuals) and excluded responses from the branches where <3 employees responded. As a result, the study's final sample was made up of 193 responses at 31 branches.

Among the respondents, 56.5% were male, and the average age was 31.8 years (*SD* = 5.94). The average working experience in banking was 7.4 years (*SD* = 6.71), and the average tenure in the current offices was 1.47 years (*SD* = 1.02). Seventy-six percent of the respondents had obtained at least a 4-year college degree, 46.1% of which majored in business.

This study was conducted in accordance with the Declaration of Helsinki and was approved by Korea University's review board.

### Measures

To conduct a multi-level study, we measured both individual-level and team-level conflict separately with different levels of references.

#### Team-level conflict

Eight items adopted from Jehn (1995) were used to measure employees' perceptions of the relationship conflict and task conflict at team level (4 items respectively). A sample item for team-level task conflict is “How often do people in your work team disagree about opinions regarding the work being done?” A sample item for team-level relationship conflict is “How much friction is there among members in your work unit?” (See Annex for details.). Cronbach's alpha was 0.93 for team-level task conflict and 0.95 for team-level relationship conflict.

In using the individual responses to analyze the team-level conflict as a characteristic of the team, *r*_*wg*_ (within-group interrater reliability)^1^ was computed for the team-level conflict measures in order to assess the extent of consensus, agreement, or within-unit variability, within a unit for the measures (James et al., 1984). The inter-rater agreement estimates were computed for team-level conflict measures; mean *r*_*wg*_ = 0.83 and median *r*_*wg*_ = 0.90 for team-level task conflict, and mean *r*_*wg*_ = 0.87, median *r*_*wg*_ = 0.89 for team-level relationship conflict. James et al. (1984) suggested that aggregation can be justified by a median agreement index of 0.70 or greater, and all of our estimates exceeded this criterion. Moreover, the ICC values for team-level task conflict [ICC (1) = 0.07, ICC (2) = 0.94] and team-level relationship conflict [ICC (1) = 0.12, ICC (2) = 0.96] were all statistically significant. Thus, these results support for the aggregation of the measures to the team level.

#### Individual-level conflict

To provide individual-level measures of conflict in teams, employees responded to eight items modified to the individual level from Jehn's (1995) team-level task conflict and relationship conflict scales (four items respectively). More specifically, to measure individual-level conflict, we asked team members to think about their individual conflict experiences with other team members rather than team unit as a whole. A sample item for individual-level task conflict is “How often do you disagree about opinions regarding the work being done with your team members?” A sample item for individual-level relationship conflict is “How much friction do you experience with your team members?” Cronbach's alpha was 0.90 for individual-level task conflict and 0.93 for individual-level relationship conflict. Because we used the same people in completing the same individual and team-based conflicts, we assessed common method bias by allowing the error terms of individual and team conflict measured by same method to be correlated; stable correlations leading us to conclude that common method bias is not a significant issue in the study (Podsakoff et al., 2003).

#### Team commitment

Team commitment was operationalized as the relative strength of an individual's identification with, and involvement in, a particular team (Mowday et al., 1982). Team commitment was measured with the 8-item scale developed by Bishop and Scott (2000) (Cronbach's alpha = 0.96). Sample items included the followings: “I talk up (brag about) this team to my friends as a great team to work on.” and “This team really inspires the very best in me in the way of job performance.”

#### Individual-level control variables

We controlled for intragroup trust, in-house training periods, and education level. Intragroup trust is considered as a positive predictor of an individual's attitude (Driscoll, 1978). Simons and Peterson (2000) and Yang and Mossholder (2004) proposed that intragroup trust plays an important role in interpreting conflict because task conflict can be easily misconstrued as being personal in nature or in motive, and thus, be erroneously categorized as relationship conflict. We measured intragroup trust using the five-item Likert-type scale used in Simons and Peterson's (2000) and the coefficient alpha was 0.92. Sample items for intragroup trust included the followings: “We absolutely respect each other's competence.” and “We expect the complete truth from each other.”

The in-house training program is a component of external compensation for employees and such compensation could be an important antecedent variable for an employee's team commitment (Porter et al., 1974). We controlled for all personal training, by asking each respondent for the total amount of in-house training they received (new employee orientation training was not included because every employee in the bank participated in this training).

#### Team-level control variables

Team size and age diversity were used in our study as team-level control variables. Team size affects the severity of conflicts between the members; when there are more employees in the group, the degree of conflict is higher (Jehn, 1995). In this study, team size was operationalized as the number of registered employees in a branch office. The influence of age diversity on team members' commitment was controlled, since Kunze et al. (2011) suggested that age diversity had a negative effect on the commitment of employees. To measure age diversity, we used the coefficient of variation (Allison, 1978) and thus, divided each team's standard deviation of age by the team's mean age.

### Analytical approach

#### Multi-level approach

Because the individual-level data were nested within team-level data, we ran HLM analyses to test the hypotheses. HLM is a statistical technique that are used for analyzing data in a clustered or “nested” structure, in which lower-level units of analysis are nested within higher-level units of analysis. For example, employees are nested within teams, which are nested within company.

In order to reduce multicollinearity, all the predictor variables were centered using the grand mean centering method. We took four steps to test the hypotheses. First, for the dependent variable (team commitment), we ran a set of null models with no predictors. Second, we then conducted level-1 analyses to determine the significance of the hypothesized individual-level antecedents (individual-level conflict) in predicting team commitment. A regression line was estimated for each of the 31 teams in this step for level-1 analysis. In the third step, level-2 analyses followed, to which we added team-level antecedents to test for additional explained variance and assess the main effects of the team-level conflict climate. In the last step, we regressed the slope estimates obtained from level-1 on the team-level predictors (team-level conflict) to detect cross-level interaction effects.

## Results

Table 1 presents the descriptive statistics and correlations for all the study variables at both the individual- and the team-level. There was a significant positive correlation between intragroup trust and team commitment (*r* = 0.52). In addition, consistent with previous research (Simons and Peterson, 2000; De Dreu and Weingart, 2003; de Wit et al., 2012), the perceptions of task and relationship conflicts in the team had strong positive correlation with each other (*r* = 0.65). To avoid multicollinearity-related problems, we ran HLM analyses for task conflict and relationship conflict respectively.

**Table 1:** Means, standard deviations, and correlations for variables.

**Individual-level Variable**	**Means**	***SD***	**1**	**2**	**3**	**4**	**5**
1. Task Conflict	4.20	1.24					
2. Relationship Conflict	3.80	1.48	0.61^**^				
3. Intragroup Trust	5.40	0.96	−0.14	−0.31^**^			
4. Inhouse Training	0.49	1.18	0.13	0.15^*^	−0.03		
5. Education	2.63	0.76	0.00	0.01	0.14	0.01	
6. Team Commitment	4.86	1.35	−0.26^**^	−0.43^**^	0.52^**^	−0.07	0.08
**Team-level Variable**	**Means**	***SD***	**1**	**2**	**3**		
1. Team size	11.42	4.32					
2. Age diversity	0.15	0.06	−0.06				
3. Task Conflict	3.81	0.80	−0.28	0.13			
4. Relationship Conflict	3.26	0.77	0.03	0.22	0.65^*^		

*Individual-Level N = 193; Team-Level N = 31*.

* *p < 0.05*,

** *p < 0.01*.

### Discriminant validity check

We performed a confirmatory factor analysis on the six variables—individual-level task conflict, individual-level relationship conflict, team-level task conflict before aggregation, team-level relationship conflict before aggregation, intragroup trust, and team commitment—to establish their discriminant validity (see Table 2). The confirmatory factor analysis with the six variables as distinct factors demonstrated good fit to the data [$\chi_{\left(362,\;\text{N}=193\right)}^{2}$ = 822.86, χ^2^/*df* = 2.27, IFI = 0.92, CFI = 0.92, RMSEA = 0.081]. Further, the results of the chi-square difference tests indicated that this four-factor model had a better fit to the data than the plausible alternative models (see Model 3 in Table 2). This six-factor model, compared with the alternative models, also provided superior point estimates for the fit measures. These results supported the discriminant validity of the six variables as distinct constructs at the individual-level.

**Table 2:** Confirmatory factor analysis of level-1 variables.

**Model Factor structure model**	**χ*^2^(df)***	**χ*^2^/df***	**IFI**	**CFI**	**RMSEA**	**RMSEA confidence interval**	**Δχ*^2^* (Δ*df*)**
1. One factor (alternative): All six scales together as one factor	3748.10(377)	9.94	0.42	0.42	0.216	0.210.222	2925.24(15)
2. Three factor (alternative): Task and relationship conflict constrained as one factor	1470.98 (371)	3.96	0.81	0.80	0.124	0.118,0.131	648.12 (9)
3. Three factor (alternative): Individual-level conflict and Team-level conflict constrained as one factor	1369.87 (371)	3.69	0.82	0.82	0.118	0.112,0.125	547.0 (9)
4. Six factor (hypothesized): ITC, IRC, TTC, TRC, intragroup trust, and team commitment as distinct factors	822.86 (362)	2.27	0.92	0.92	0.081	0.074,0.089	–

*N = 193, All χ^2^ values are significant at p < 0.05. IFI, incremental fit index; CFI, comparative fit index; RMSEA, root-mean-square error of approximation; ITC, individual-level task conflict; IRC, individual-level relationship conflict, TTC, team-level task conflict; TRC, team-level relationship conflict. All the nested chi square difference tests reported above are significant at p < 0.05. We decide to go on with the last model because it as the most parsimonious model*.

### Hypotheses testing

#### Result of HLM null model

We followed the HLM procedure recommended by Hoffmann (1997) to test our hypotheses. Our hypotheses predict that both individual-level and team-level variables would have a significant negative relationship with the individual employees' team commitment. In order for these hypotheses to be tested, there should be significant between-team variance in individual team commitment. A chi-square test showed that the between-team variance in team commitment was significant [χ^2^_(30)_ = 112.84, *p* < 0.001]. Based on this result from testing the null model, the next analysis was performed.

#### Individual-level conflict

We estimated the level-1 model that did not include team-level conflict. We predicted that individual-level task conflict (Hypothesis 1a) and relationship conflict (Hypothesis 1b) would be negatively associated with an employee's team commitment. The results indicate that individual-level relationship conflict was negatively associated with team commitment (γ = −0.24, *p* < 0.01), which supports Hypothesis 1b (see Tables 3, 4). However, contrary to the predictions of Hypothesis 1a, individual-level task conflict was negatively, but not significantly related to the team commitment (γ = −0.16, *p* > 0.10).

**Table 3:** HLM results for the effects of task conflict on team commitment.

**Variables**	**Control variable**		**Individual-level predictors**		**Team-level predictors**		**Cross level predictors**	
	**γ**	***SE***	**γ**	***SE***	**γ**	***SE***	**γ**	***SE***
**INDIVIDUAL- LEVEL PREDICTORS**								
Intercept	4.82^***^	0.16	4.88^***^	0.11	4.88^***^	0.11	4.90^***^	0.11
Intragroup Trust	0.67^***^	0.09	0.67^***^	0.10	0.66^***^	0.10	0.65^***^	0.10
In-house Training	−0.08	0.08	−0.05	0.06	−0.03	0.08	−0.02	0.08
Education	0.08	0.10	−0.01	0.09	−0.03	0.09	−0.03	0.09
Task Conflict (Hypothesis 1a)			−0.16	0.11	−0.09	0.12	−0.10	0.12
**TEAM-LEVEL PREDICTORS**								
Team size	0.04	0.03	0.03	0.02	0.02	0.02	0.02	0.02
Age Diversity	−0.72	2.19	−0.07	1.25	0.12	1.21	0.24	1.22
Team-level Task Conflict (Hypothesis 2a)					−0.28^*^	0.12	−0.33^*^	0.13
**CROSS-LEVEL INTERACTIONS**								
Team-level X Individual-level								
Task Conflict (Hypothesis 3a)							−0.09	0.09
Model Deviance	583.81		536.07		532.04		535.9641.38	

*Individual-Level N = 193; Team-Level N = 31*.

* *p < 0.05*,

*** *p < 0.001*.

**Table 4:** HLM results for the effects of relationship conflict on team commitment.

**Variables**	**Control variable**		**Individual-level predictors**		**Team-level predictors**		**Cross level predictors**	
	**γ**	***SE***	**γ**	***SE***	**γ**	***SE***	**γ**	***SE***
**INDIVIDUAL- LEVEL PREDICTORS**								
Intercept	4.82^***^	0.16	4.84^***^	0.11	4.85^***^	0.10	4.90^***^	0.09
Intragroup Trust	0.67^***^	0.09	0.56^***^	0.10	0.55^***^	0.10	0.54^***^	0.10
In-house Training	−0.08	0.08	−0.05	0.07	−0.05	0.07	−0.02	0.06
Education	0.08	0.10	0.11	0.11	0.09	0.11	0.10	0.11
Relationship Conflict (Hypothesis 1b)			−0.24^**^	0.08	−0.18^*^	0.08	−0.20^*^	0.08
**TEAM-LEVEL PREDICTORS**								
Team size	0.04	0.03	0.01	0.02	0.02	0.02	0.01	0.02
Age Diversity	−0.72	2.19	−0.65	1.34	−0.14	1.30	0.40	1.26
Team-level Relationship Conflict (Hypothesis 2b)					−0.43^**^	0.12	−0.43^***^	0.12
**CROSS-LEVEL INTERACTIONS**								
Team-level X Individual-level								
Relationship Conflict (Hypothesis 3b)							−0.15^*^	0.07
Model Deviance	538.81		539.62		531.86		533.33	

*Individual-Level N = 193; Team-Level N = 31*.

* *p < 0.05*,

** *p < 0.01*,

*** *p < 0.001*.

#### Adding team-level conflict

To test Hypothesis 2a and 2b, we developed an HLM model in which the individual-level conflict variables were the level-1 predictors and then regressed the intercept coefficients obtained from level-1 on the measures of team-level conflict at level-2. As reported in Tables 3, 4, both team-level task conflict (γ = −0.28, *p* < 0.05) and relationship conflict (γ = −0.43, *p* < 0.01) demonstrated significant relationships with employee team commitment after we had accounted for individual-level conflicts. Hence, Hypothesis 2a and 2b were supported.

#### Testing cross-level interaction

We predicted that the relationship between individual-level conflict and an employee's team commitment would be moderated by team-level conflict such that the negative relationship would be stronger when there is a high level of team-level conflict (Hypothesis 3a and 3b).

A prerequisite for testing these cross-level interactions is that there should be significant random variance for the individual-level conflict variables in the intercepts-as-outcomes models estimated in the previous step (Liao and Chuang, 2004). There was significant random variance in the slopes for individual-level task conflict (τ_22_ = 0.21, *p* < 0.01) and relationship conflict (τ_22_ = 0.14, *p* < 0.01), indicating the possible presence of team-level moderators. With the prerequisites fulfilled, we introduced team-level conflict as a level-2 moderator.

**Figure 2:**
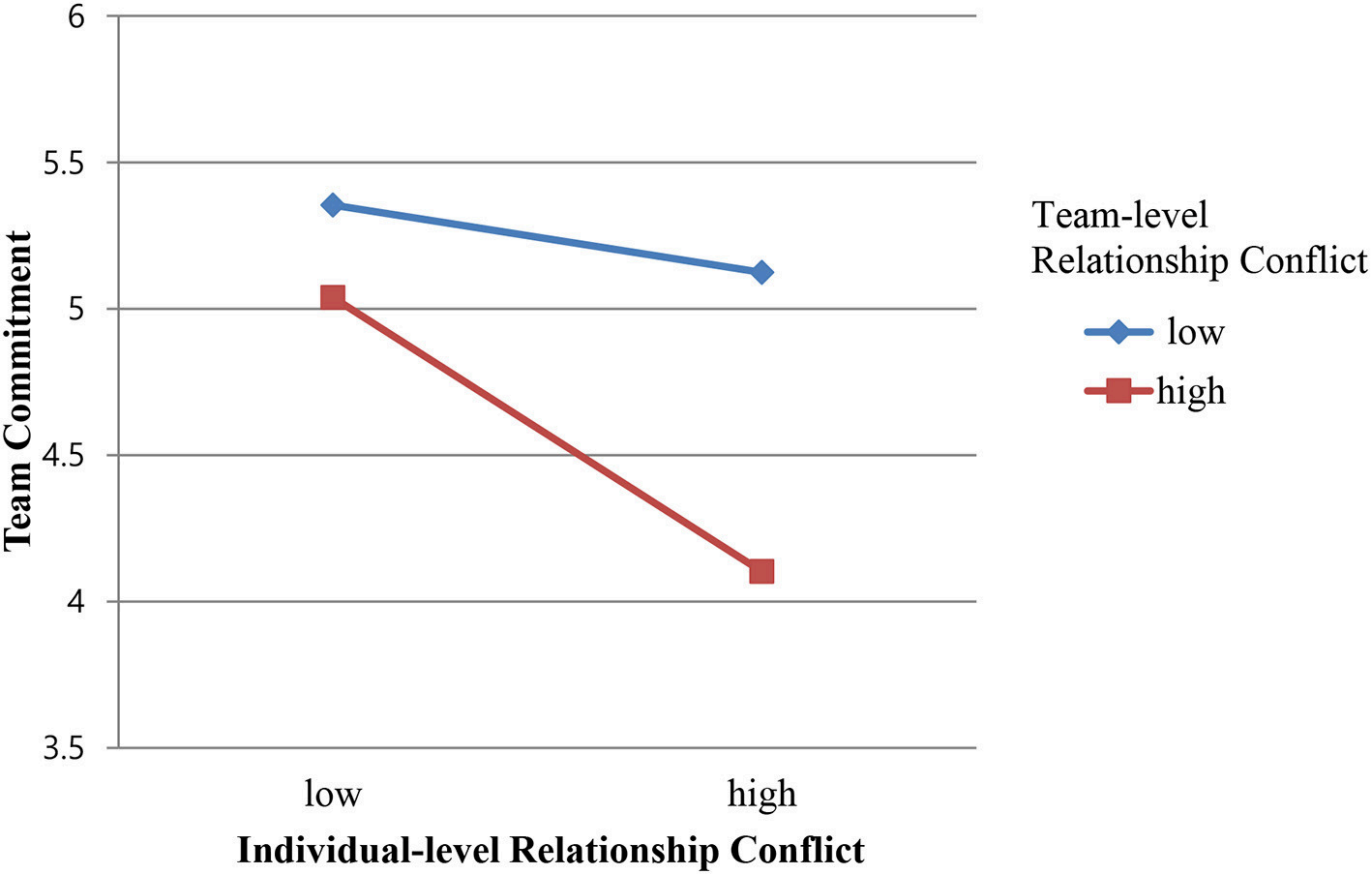
Plot of cross-level moderating effects of team-level relationship conflict on the relationship between individual-level relationship conflict and team commitment.

Hypothesis 3a was not supported because there was no significant interaction between individual-level and team-level *task* conflict (γ = −0.09, *n.s*.). However, the results indicate that the interaction of individual-level and team-level *relationship* conflict was significant (γ = −0.15, *p* < 0.05), supporting Hypothesis 3b. We then tested whether this interaction effect was aligned with the hypothesized trend, utilizing a simple slope test at cross-level interaction (Preacher et al., 2006). The interaction plot (Figure 2) graphically represents cross-level moderation, showing the relationship between individual-level relationship conflict and employee team commitment in teams with (1) high and (2) low relationship conflict climates. As expected, individual-level relationship conflict had a stronger association with team commitment when team-level relationship conflict was high (+1 SD) rather than low (−1 SD). The results indicated that the simple slope at +1 SD of team-level relationship conflict was negatively significant (*B* = −0.59, *t* = −2.28, *p* < 0.05), whereas the simple slope at −1 SD of team-level relationship conflict was not significant (*B* = −0.25, *t* = −1.12, *p* > 0.05). Summarizing the result, the interaction plot revealed that team-level relationship conflict intensified the negative relationship between individual-level relationship conflict and the team commitment of the team members.

## Discussion

In this study, we aimed to distinguish and verify two levels of conflict (i.e., individual- and team-level). Then we investigated how two levels of conflict and two types of conflict (relationship conflict and task conflict) influence team commitment. More specifically, we examined the combined effect of individual-level and team-level conflict on individual attitudes toward the team (i.e., team commitment) using a multi-level perspective (Klein and Kozlowski, 2000; Ceschi et al., 2014).

First, we found that individual-level relationship conflict was significantly and negatively related to team members' team commitment. Even though team commitment is an important factor for team success, there has been a paucity of studies empirically testing these relationships. The negative association between individual-level relationship conflict and team members' team commitment is consistent with the findings of previous studies that conducted individual-level analysis of intragroup conflict (Jehn, 1995; Jehn et al., 1999; de Wit et al., 2012).

However, we found that individual-level task conflict did not significantly relate to team commitment. One possible explanation for this insignificant relationship is that the two opposing mechanisms were operative at the same time. On one hand, self-verification theory (Swann et al., 2004) posits that individuals are less likely to be committed to the team when their opinions are challenged by other group members, since it can be interpreted as his or her inability or incompetence. On the other hand, according to self-perception theory (Bem, 1967, 1972), individuals tend to infer their attitudes from their own behavior in the same way that an outside observer might. Therefore, individuals who experience task conflict or disagreement with other members may judge themselves to be highly involved in the work and committed to the team. In other words, employees can infer that they are involved in the task conflict with other members because they have high motivation toward team tasks. Therefore, experiencing task conflict can be considered to be a social cue or signal that an individual is committed to the team. In addition, they can be satisfied because they had a chance to voice their opinions on the task at hand (Simons and Peterson, 2000). Thus, the insignificant result might reflect the trade-off between the negative and positive effects of individual-level task conflict on team commitment.

Second, we found that team-level task and relationship conflicts had negative relationships with team commitment even after controlling for individual-level task and relationship conflicts. By differentiating the two levels of conflict, we were able to show the incremental effects of team-level conflict on team commitment. Previous studies have not paid attention to the two different levels of intragroup conflict: individual-level and team-level. Using a multi-level approach, our study implies that team-level conflict may have incremental effects on team commitment beyond the effects of individual-level conflict. The negative relationship between team-level conflict (both task and relationship conflict) and the team commitment of employees is consistent with previous empirical studies on team conflict focusing on team level analysis. For example, Bailey (2000) found that conflict at the team-level decreased employee satisfaction with team. Interestingly, although we found that individual-level task conflict was not significantly related to team commitment, team-level task conflict had a negative impact on team commitment. These results suggest that once an individual is already involved in an individual-level task conflict, it can be a signal that he or she is committed to the team. However, when other group members are in a team-level task conflict (regardless the focal individual may or may not be involved in the task conflict), individuals will not be committed to the team in order to avoid conflict. In addition to the CFA results for the discriminant validity, this finding implies that team-level task conflict is a distinct construct that is different from individual-level task conflict.

Finally, by examining the cross-level interactions between the conflicts at the two levels, we found that a high degree of team-level relationship conflict aggravated the negative relationship between individual-level relationship conflict and team commitment. We theorized and tested the cross-level interactions between team-level and individual-level conflicts on team commitment. While previous studies have investigated interactions between the two types of conflict (e.g., task and relationship conflict) (Eisenhardt et al., 1997; Mooney et al., 2007), the conflict literature has not paid attention to these cross-level interactions partly because it has not paid specific attention to the level issue in conflict. Based on social information theory, we proposed that team-level conflict aggravated the negative relationship between conflict and team commitment. Also, we found that team-level relationship conflict moderated the relationship between individual-level relationship conflict and team commitment such that the negative relationship was stronger when team-level relationship conflict was prevalent. This result suggests that immediate social context (i.e., team-level relationship conflict) serves as an important factor that influences how individual-level relationship conflict reflect on employee attitudes toward the team.

In contrast, with regard to task conflict, there were no cross-level interactions between the two levels. The different results between the two types of conflict can be explained by the social information processing perspective of Salancik and Pfeffer (1978). In particular, Salancik and Pfeffer (1978) suggest that as information becomes increasingly ambiguous, individuals rely more on social comparisons to assess it. Compared to the relationship conflict information, task conflict information is more certain and less ambiguous because task conflict is dependent on task contents and perceived more objectively. Thus, when experiencing individual-level task conflict, individuals may rely less on cues from their surrounding environments in interpreting conflict information. Conversely, relationship conflict information is more uncertain and ambiguous because it tends to be more interpersonal and emotional. Therefore, the social information processing is more likely to occur in relationship conflict information processing, and as a result individuals are more affected by the social context in determining their attitude toward their team. Future studies should focus on investigating the mechanisms that drive this interesting finding.

### Implications for practice

This paper provides interesting implications for practice. In order to make employees be more committed to the organization, managers should try to minimize relationship conflict regardless of its level (individual or team). However, this paper provides different prescriptions for dealing with different levels of task conflict (individual and team level). First, team-level task conflict should be minimized since individuals do not want to become embroiled in a dispute with their team members. That is, employees want to avoid factional activities. In contrast, to some extent, individual-level task conflict can be recommended since it does not reduce team commitment of employees. It can be a signal that team members are committed to the team. Furthermore, the satisfaction of employees could increase because they have opportunities to express their opinions regarding the task.

This suggests that team leaders who are concerned with improving the commitment of their team members should not only differentiate between task conflict and relationship conflict but also distinguish between individual-level and team-level conflict. With regard to task conflict, it is important for team leaders to resolve conflict at team-level such as task coordination in teams. However, with regard to relationship conflict, dealing with conflict at team-level is not sufficient. It is required for team leaders to know who have the relationship conflict and to deal with conflict personally.

## Limitations

Our study has several limitations. First, there can be a generalizability issue because this study used Korean samples only. In Korea, conflict is less accepted than in Western countries because the traditional corporate culture of South Korea is based on Confucianism that emphasizes harmonious relationship with others. However, the result of our study would be even stronger in Western societies where conflict is generally more accepted.

Second, we measured individual-level and team-level conflict variables from same person, a practice that may introduce common method bias (Podsakoff et al., 2003). In the future, more objective data that are focused on team-level conflict should be examined.

Third, we used only employee team commitment as a dependent variable and measured team commitment using a self-report. In order to generalize our findings to team literature, future studies need to use objective performance measures in order to test the distinct effects of team-level and individual-level conflicts on team performance.

Another limitation of our study is that the sampled branches were not identical in terms of team size. However, we believe that this limitation does not affect the interpretation of the result not only because the branches belonged to the same organization, but also because we controlled for the team size effect and any other team-dependent effect with the HLM analysis.

Finally, this study showed that there was no significant relationship between the individual-level task conflict and team commitment. However, task conflict can be transformed into emotional relationship conflict as time passes (Simons and Peterson, 2000; De Dreu and Weingart, 2003; Yang and Mossholder, 2004). Because this study used only cross-sectional data, the conflicts were measured at a certain point of time. Therefore, future studies may need to include longitudinal data to trace how a certain type of conflict changes into another type of conflict across time. We can then investigate the dynamic relationship between the two different types of conflict at both individual and team levels.

## Conclusion

We found that team-level conflict, either relationship or task, had an incremental effect on individual members' team commitment. In addition, while individual-level task conflict had a non-significant negative influence on team commitment overall, team-level task conflict had a negative influence on team members' team commitment. Further, we found team-level relationship conflict aggravated the negative relation between individual-level conflict and team commitment. These findings expand our understanding of team conflict by revealing that team-level conflict is distinct from individual-level conflict. This implication encourages future studies to investigate the respective roles of individual- and team-level conflicts separately in team dynamics, and their interplay for team success. As a practical implication, managers should pay attention not only to individual-level relationship conflict, but also to team-level relationship conflict because a high level of team-level conflict may aggravate the negative influence of individual-level conflict on team commitment and performance.

## Author contributions

SL wrote the first draft and edited by SK. MK analyzed data with SL. SL, SK, MK, SS, and I-JP generated idea for this study.

### Conflict of interest statement

The authors declare that the research was conducted in the absence of any commercial or financial relationships that could be construed as a potential conflict of interest. The reviewer, AC and handling Editor declared their shared affiliation.

## Footnotes

^1^r_wg_ = 1 − $\left(S_{\text{xj}}^{2}/\sigma_{\text{EU}}^{2}\right)$. r_wg_ is the within-group interrater reliability for a group of K judges on a single item X_j_, ${\text{S}}_{\text{xj}}^{2}$ is the observed variance on X_j_, and $\sigma_{\text{EU}}^{2}$ is the variance on X_j_ that would be expected if all judgments were due exclusively to random measurement error.

## Annex

Measurement of individual-level conflict and team-level conflict (task conflict).

**Table:**

Item 1	Individual-level	How often do you disagree about opinions regarding the work being done with your team members?
	Team-level	How often do people in your work team disagree about opinions regarding the work being done?
Item 2	Individual-level	How frequently do you experience conflicts about ideas with your team members?
	Team-level	How frequently are there conflicts about ideas in your work unit?
Item 3	Individual-level	How much conflict about work do you experience with your team members?
	Team-level	How much conflict about work is there in your work unit?
Item 4	Individual-level	To what extent do you experience differences of opinion with your team members?
	Team-level	To what extent are there differences of opinion in your work unit?

(Relationship Conflict)

**Table:**

Item 1	Individual-level	How much friction do you experience with your team members?
	Team-level	How much friction is tdere among members in your work unit?
Item 2	Individual-level	How much personality conflicts do you experience with your team members?
	Team-level	How much are personality conflicts evident in your work unit?
Item 3	Individual-level	How much tension do you experience with your team members?
	Team-level	How much tension is there among members in your work unit?
Item 4	Individual-level	How much emotional conflict do you experience with your team members?
	Team-level	How much emotional conflict is there among members in your work unit?
